# Development and Application of a LC-MS/MS Method for Simultaneous Quantification of Four First-Line Antituberculosis Drugs in Human Serum

**DOI:** 10.1155/2020/8838219

**Published:** 2020-07-08

**Authors:** Yunliang Zheng, Nana Xu, Xingjiang Hu, Qiao Zhang, Yanpeng Liu, Qingwei Zhao

**Affiliations:** ^1^Zhejiang Provincial Key Laboratory for Drug Evaluation and Clinical Research, The First Affiliated Hospital of Zhejiang University, School of Medicine, 79 Qingchun Road, Hangzhou, Zhejiang 310003, China; ^2^Research Center of Clinical Pharmacy, The First Affiliated Hospital of Zhejiang University, School of Medicine, 79 Qingchun Road, Hangzhou, Zhejiang 310003, China

## Abstract

A simple, rapid, and sensitive liquid chromatography (LC)/mass spectrometry (MS) method was established and validated for simultaneous quantitation of pyrazinamide, isoniazid, rifampicin, and ethambutol in human blood sample. Samples were pretreated by a single-step precipitation with acetonitrile. Chromatographic separation was achieved on XSelecT HSS T3 column by gradient elution with a total run time of 5.0 min. MS detection was performed by a triple quadrupole tandem mass spectrometer in the multiple reaction monitoring mode with a positive electrospray ionization source. Isotope-labeled internal standard, especially rifampicin-D8, was applied to adjust for the loss during sample treatment. The established LC-MS/MS method showed a wide analytical range (pyrazinamide: 1.02∼60.0 *μ*g/mL, isoniazid: 0.152∼10.0 *μ*g/mL, rifampicin: 0.500∼30.0 *μ*g/mL, and ethambutol: 0.0998∼5.99 *μ*g/mL) and a good linearity (*r* > 0.99 for the four analytes) with acceptable accuracy and precision (90.15%∼104.62% and 94.00%∼104.02% for intra- and interaccuracy, respectively; RSD%: <12.46% and <6.43% for intra- and interprecision, respectively). It also showed excellent recoveries (79.24%∼94.16% for all analytes) and absence of significant matrix effect. This method was successfully applied to the quantification of four first-line antituberculosis (anti-TB) drugs, suggesting its suitability for therapeutic drug monitoring in the clinical practices.

## 1. Introduction

Tuberculosis (TB) is an ancient disease, but it is still one of the top ten causes of death, which kills 1.5 million people every year all over the world [1, 2]. A long-term treatment with multiple drugs is necessary for TB patients [3], which was proposed as follows [4]: an intensive phase with four first-line antituberculosis drugs (pyrazinamide (PZA), isoniazid (INH), rifampicin (RFP), and ethambutol (EMB)) for the first two months, then followed by a continuation phase with isoniazid and rifampicin for the next four months. Although this regimen is highly effective for most drug-susceptible TB patients, some patients do not respond adequately and even suffer from treatment failure and drug resistance [5].

Numerous factors have been reported to be associated with poor response. Drug exposure, like serum concentration level, is of vital importance. Lower serum concentration of antituberculosis drugs results in treatment failure and drug resistance [6], especially for patients with concomitant diseases, like HIV, type 2 diabetes [7], and gastrointestinal tract problems, because they are more likely to suffer from a poor drug absorption and drug-drug interaction. In addition, some adverse drug effects, such as hepatic dysfunction and thrombocytopenia, will develop more frequently with increasing serum drug levels [8]. Therapeutic drug monitoring (TDM) has been proposed as an effective strategy to help clinics make instant dosage adjustment of anti-TB drugs for patients with poor response [9]. However, it has not been widely applied in clinical practice for lack of effective detection methods. To simultaneously measure serum concentrations of four first-line anti-TB drugs, a simple and robust method is urgently warranted.

Some chromatography-based methods such as HPLC-ultraviolet detection, HPLC-fluorescence detection, and HPLC-tandem mass spectrometry (MS/MS) have been introduced. However, all of these methods have their own disadvantages, like a long time for chromatographic separation, complicated sample pretreatment, simultaneous quantification of only one to three drugs, and using homologues or other drugs as an internal standard [10–15]. Although some studies chose rifampicin-D3 as an isotope-labeled internal standard [16–18], our preexperiment indicated that rifampicin-D3 existed in the rifampicin standard, which might interfere with the quantification.

Therefore, this study aimed to establish a simple and reliable LC-MS/MS method to simultaneously quantify four first-line anti-TB drugs (pyrazinamide, isoniazid, rifampicin, and ethambutol) with rifampicin-D3 as an internal standard, thus improving its applicability in clinical practice.

## 2. Materials and Methods

### 2.1. Instruments

Liquid chromatography (LC) system (AB Sciex Jasper HPLC) was coupled with mass spectrometer (MS) system (AB Sciex Triple Quad 4500). AB Sciex Jasper HPLC consisted of a binary pump, an autosampler, a column oven, and a controller. AB Sciex Triple Quad 4500MD (Applied Biosystems Sciex, Ontario, Canada) was operated in positive ion mode with electrospray ion (ESI) source, using multiple reaction monitoring (MRM). Analyst software version 1.6.3 (Sciex, Toronto, Canada) was used for system control, data acquisition, and processing.

Electronic balance (MettlerToledoXP26, *d* = 0.001 mg, Switzerland), vortex oscillator (Ql-901, Jiangsu, China), high-speed low-temperature centrifuge (Eppendorf5417c, Germany), and Milli-Q pure water system (Millipore, USA) were also used.

### 2.2. Chemicals and Reagents

**Figure 1:**
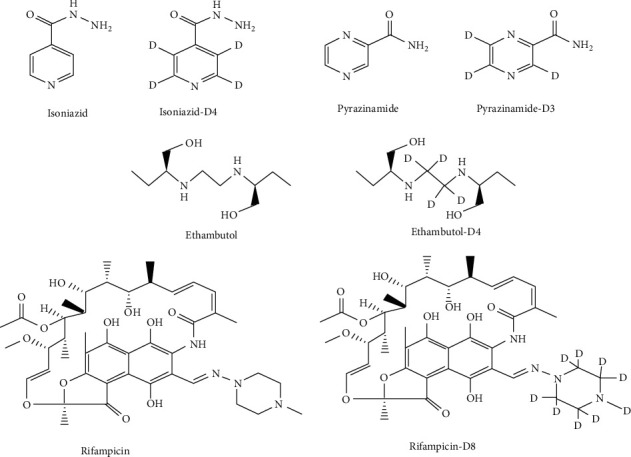
Molecule structures of the four analytes and ISs.

Pyrazinamide (lot: 100178–201104), isoniazid (lot: 100578–201502), rifampicin (lot: 130496–201403), and ethambutol dihydrochloride (lot: 100165–201705) were purchased from National Institutes for Food and Drug Control. Internal standards (ISs), pyrazinamide-D3 (lot: CRC-OW3-056), isoniazid-D4 (lot: 2-JLO-114-1), rifampicin-D3 (lot: 5-YEN-103-103-1), rifampicin-D8 (lot: NJ-07222019), and ethambutol-D4 (lot: 1-WEW-51-4), were purchased from Toronto Research Chemicals, Canada. The purities of these analytes were all above 98.0%. Molecule structures of the four analytes and corresponding ISs are presented in Figure 1.

Methanol (HPLC grade) and acetonitrile (HPLC grade) were purchased from Merck Drugs & Biotechnology, German. Trifluoroacetic acid (HPLC grade) was purchased from Sigma Aldrich Fluka, USA. Ammonium formate (HPLC grade) was purchased from Anaqua Chemical Supply, USA. Water was prepared by a Milli-Q deionization system (Millipore, Billerica, MA, USA). Blank human serum was obtained from the physical examination population of The First Affiliated Hospital, Zhejiang University School of Medicine.

### 2.3. Chromatographic Conditions

Chromatographic separation was accomplished by a XSelecT HSS T3 column (3.0 × 100 mm, 2.5 *μ*m, Waters, USA) at a column temperature of 40°C. The mobile phases consisted of solvent A (5 mM ammonium formate and 0.1% trifluoroacetic acid in water) and solvent B (5 mM ammonium formate and 0.1% trifluoroacetic acid in methanol) mixed in proper proportions. A gradient elution was programmed: 0-2 min 5% ⟶ 95% solvent B; 2–4 min 95% solvent B; 4–4.1 min 95%⟶5% solvent B; 4.1–5 min 5% solvent B. The total run time is 5 min at a flowing rate of 0.40 mL/min, and the injection volume was 1 *µ*L.

### 2.4. Mass Spectrometry Conditions

Ionization was achieved using heated electrospray ionization (HESI) with a spray voltage of 4000 V for positive mode and 3000 V for negative mode.

MS conditions for target drugs and internal standards were optimized by delivering a single solution containing 1 *μ*g/mL PZA, INH, EMB, or RFP into the MS system using a syringe pump at a constant flow rate of 10uL/min. Mass spectrometer was operated in positive ion mode with electrospray ion (ESI) source. Firstly, the precursor ions were determined by Q1 full scan, and product ions were selected by product ion scan for the subsequent transition. Then, MS parameters associated with collision-induced dissociation were optimized by multiple reaction monitoring (MRM) scan. Finally, MRM transitions of the highest sensitivity and minimum interference were determined for quantification. The transitions and optimized parameters for each analyte are listed in Table 1. The dwell time of each MRM transition was set at 100 ms. The source parameters were set as following: ion spray voltage (IS): 5000 V, ionization source temperature: 550°C, curtain gas: 25 psi, ion source gas 1: 60 psi, ion source gas 2: 60 psi, and collision gas: 6 psi.

### 2.5. Preparation of Solutions and Samples

Stock solutions of pyrazinamide 1.20 mg/mL, isoniazid 1.41 mg/mL, rifampicin 1.35 mg/mL, and ethambutol 1.05 mg/mL were prepared by dissolving each powered compound in pure methanol and stored at −20°C. Working solutions were obtained by mixing four stock solutions and then diluting in 50% methanol to seven levels from high to low concentrations. Calibrators at seven levels (calibrator A-G) were finally prepared by spiking each working solution (5 *μ*L) into blank human serum (50 *μ*L). Quality control samples at high, medium, and low concentrations were prepared in the same way as calibrators. The concentrations of calibrators and QC were chosen in accordance with Chinese Pharmacopoeia and were summarized in the supplement.

Internal standard stock solutions of 1.0 mg/mL INH-D4, RIF-D8, EMB-D4, and PZA-D3 were separately prepared with pure methanol and stored at −20°C. Internal standard working solution was made by mixing and diluting internal standard stock solutions with 50% methanol to 10.0 *μ*g/mL INH-D4, 1.0 *μ*g/mL EMB-D4, 25.0 *μ*g/mL RFP-D8, and 100.0 *μ*g/mL PZA-D3, separately.

### 2.6. Samples Treatment

Under yellow light, 50 *μ*L serum sample and 5 *μ*L internal standard working solution were transported into 1.5 mL centrifuge tube. 150 *μ*L acetonitrile were added as protein precipitant. The mixed solutions were vibrated for 30 seconds. Then, they were centrifuged for 10 min with a speed of 14000 rpm at 4°C. Finally, 150 *μ*L supernatants were transferred into an autosampler vial (with insert) for detection.

### 2.7. Methodological Validation

The method was validated following Chinese Pharmacopoeia for bioanalytical method validation in terms of selectivity, precision, accuracy, linearity, calibration curve, lower limit of quantification (LLOQ), carryover, and stability. Matrix effect and recovery were also evaluated.

#### 2.7.1. Selectivity

Double blank solution (DB) was prepared by mixing blank human serums from 10 people of physical examination. Single blank solution (B) was prepared by adding 5 *μ*L internal standard working solution to 50 *μ*L blank human serum. Besides, LLOQ was also used. These three solutions were pretreated in the same method as described in Samples Treatment section and then detected by LC-MS/MS system. Chromatographic peak and retention time of each analyte and/or internal standard in DB, B, and LLOQ were recorded and compared. If peak area of endogenous or exogenous interferents in DB solution was less than 20% of that in LLOQ and 5% of all ISs, a high selectivity was demonstrated.

#### 2.7.2. Linearity and Lower Limit of Quantification (LLOQ)

Seven calibrators were pretreated by the method mentioned in Samples Treatment section and analyzed by LC-MS/MS system in three separate analytical runs within two days. In each analytic run, calibration curve was plotted with concentrations of the analyte as *X*-axis, peak area ratios of the analyte to internal standard as *Y*-axis, and 1/*X*^2^ as weight factor. Linearity was evaluated by correlation coefficient (*R*). If *R* is higher than 0.99, linearity within analysis range proves good.

LLOQ was determined when the ratio of *S*/*N* was above 10 and the accuracy and precision were within the acceptance criteria (calculated values were ±20% of the theoretical value and RSD <20%).

#### 2.7.3. Carryover

Carryover was evaluated by injecting DB after upper limit of quantification (ULOQ). If the peak area of analyte in DB was lower than 20% of the LLOQ and 5% for the internal standard, no carryover effect existed.

#### 2.7.4. Precision and Accuracy

Within-run precision and accuracy were assessed in QC samples at three different levels (low, medium, and high concentrations) and LLOQ with six replicates. Between-run accuracy and precision were assessed using QCs and LLOQ from three runs analyzed on two different days. Calibration standards were along with every run and calibration curve was plotted for concentration calculation. Accuracy was expressed as the percentage bias between the actual and measured value. Precision was represented by RSD. According to the guideline, RSD should be below 15% and percentage bias should be between 85%∼115% for QCs, while RSD <20% and percentage bias should be within 80%∼120% for LLOQ.

#### 2.7.5. Recovery and Matrix Effect

Method recovery and matrix effect were measured in QC samples at three levels (low, medium, and high concentrations) with five replicates. Recovery was determined by comparing the peak area of analyte or internal standard spiked before extraction to the peak area of analyte or internal standard spiked after extraction. Matrix effect was evaluated by comparing the normalized peak area ratio of analyte to internal standard spiked after extraction (B) to those dissolved with pure solutions (acetonitrile).

#### 2.7.6. Stability

Stability of the analytes in serum was investigated in QC samples at three levels in four replicates. Stability was evaluated after they were stored at room temperature for four hours, autosampler room (10°C) for four days, −70°C refrigerator for 30 days, or after three freeze-thaw cycles (−70°C ⟶ room temperature).

### 2.8. Method Application to Clinical Practice

This method was applied to monitor serum concentrations of four first-line antituberculosis drugs for tuberculosis patients. These patients all came from the tuberculosis ward of the First Affiliated Hospital, Zhejiang University School of Medicine. Their blood samples were collected at 0 h (before treatment) and 2 h and 6 h after taking the medicines.

This study was conducted in accordance with basic ethical principle. Only abandoned human serums from physical examination population was used for method development. For method application, patient samples were collected according to the doctors' orders. This study was approved by the Ethics Committee of The First Affiliated Hospital, Zhejiang University School of Medicine. The approval letter (2020IIT41) was also obtained from the Ethics Committee.

## 3. Results

### 3.1. Selectivity

**Figure 2:**
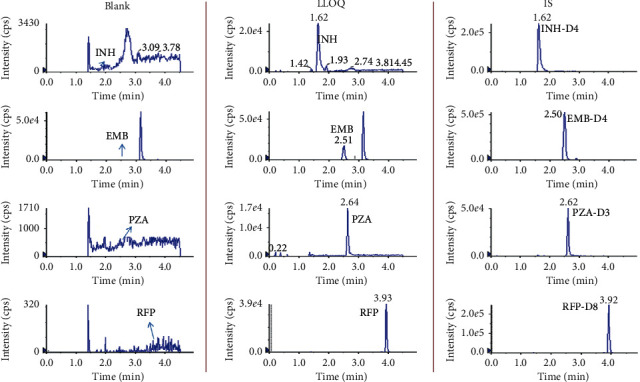
Chromatograms of blank human serum, LLOQ, and serum sample with ISs.

The chromatograms of DB, B, and LLOQ samples are shown in Figure 2. No interfering peaks at specific retention times of analytes of interest were observed in DB and B, showing an excellent selectivity.

### 3.2. Calibration Curve and LLOQ

Calibration curve was plotted by the peak area ratio of analyte and internal standard as *Y*-axis and concentration of the analyte as *X*-axis. Regression equations and correlation coefficients of each analyte were calculated by integrating the results from three runs. Good linearity was exhibited for each analyte (*r* > 0.99). LLOQ for each analyte was determined according to the ratio of *S*/*N* ≥ 10, together with intra- and interaccuracy and precision. Regression equations, correlation coefficients, linearity range, and LLOQ are summarized in Table 2.

### 3.3. Carryover

ULOQ and DB sample were injected into LC-MS/MS system in succession, and no interferant peak appeared at the specific retention times of the analytes of interests in the chromatogram of DB. Therefore, carryover was absent for all analytes.

### 3.4. Intra- and Interprecision and Accuracy

As shown in Table 3, this method exhibited a high accuracy and precision. For intraprecision and accuracy, RSDs were all below 12.46% and accuracies were all between 90.15%∼104.62% for the four analytes at each level. For interprecision and accuracy, they were also within the acceptance criteria with RSDs <8.49% and accuracies were between 94.00% and 104.02%.

### 3.5. Recovery and Matrix Effect

The results of recovery and matrix effect are summarized in Table 4. Recoveries varied from 79.24% to 94.16% for the four analytes and showed little variation at different QC levels. The normalized matrix effects varied between 0.87 and 1.05, which were also consistent at different QC levels. Neither ion suppression nor ion enhancement was found for any analytes.

### 3.6. Stability

All the four analytes were stable after storing in autosampler (10°C) for four days (accuracy: 93.17% to 105.67%), in −70°C refrigerator for 30 days (accuracy: 86.53% to 110.25%), and after three cycles of freezing in −70°C to thawing in room temperature (accuracy: 90.3% to 100.2%).

When stored at room temperature for four hours, INH showed a degradation trend (accuracy: 78.35% at low level and 83.58% at high level), and the other three analytes were stable (accuracy ranging from 89.7% to 108.25%).

**Figure 3:**
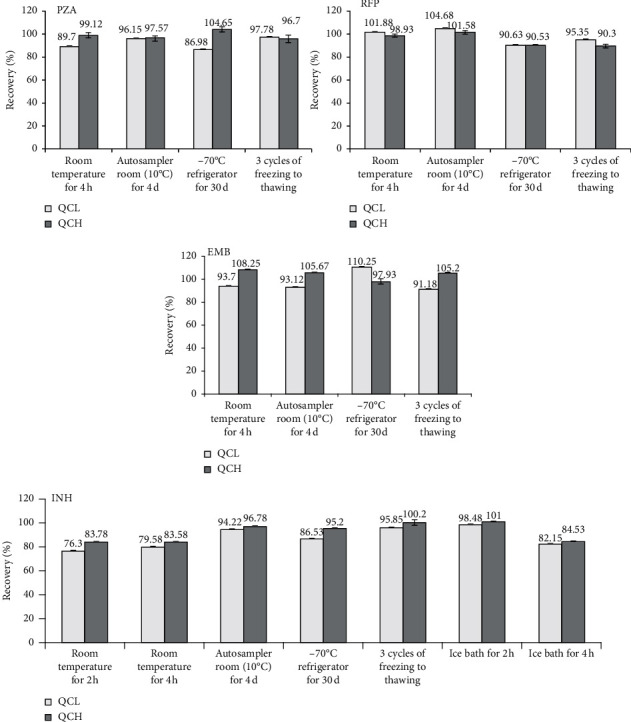
Stability of four analytes in QC samples of two levels after storing in several conditions (*n* = 4).

Therefore, stability of INH in ice bath condition was further explored. When placed in ice bath for 4 h, INH was still unstable (accuracy 82.15% at low level and 84.53% at high level). However, it was stable in an ice bath for 2 h (accuracy 98.48% at low level and 101% at high level). Details are presented in Figure 3.

### 3.7. Application to Clinical Samples

**Figure 4:**
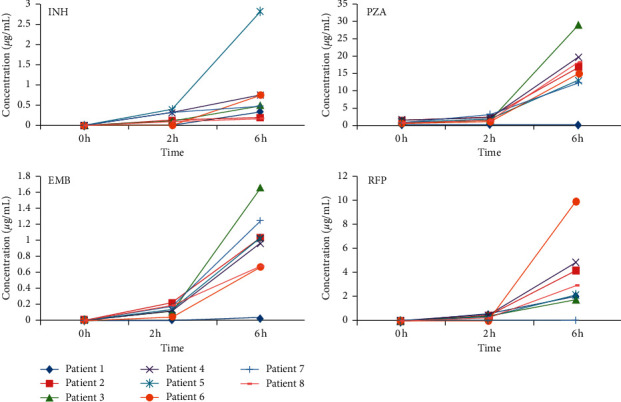
Serum concentrations of four first-line antituberculosis drugs at 0 h, 2 h, and 6 h from eight patients.

Serum drug concentrations of the four first-line antituberculosis agents at 0 h, 2 h, and 6 h for eight patients were monitored using the established method in this study. As shown in Figure 4, serum drug concentrations increased with time, and all patients reached the highest concentration at 6 h.

## 4. Discussion

### 4.1. Optimization of Chromatographic Conditions

**Figure 5:**
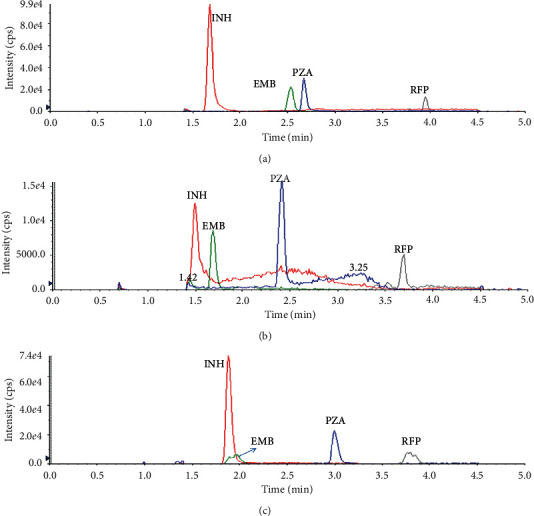
Chromatograms of the selected columns: (a) XSelecT HSS T3 column; (b) Waters XBridge Shield RP18 column; (c) Agilent Zorbax SB-C18.

In order to acquire an optimal chromatographic separation and good peak shape, various composition (formic acid, ammonium acetate, methanol, and acetonitrile) and rations of the mobile phases were tested on the XBridge Shield RP18 column (3.0 × 100 mm, 2.5 *μ*m); water and methanol with 5 mM ammonium formate and 0.1% trifluoroacetic acid were separately determined to be the most appropriate mobile phase. Then, several HPLC columns including Waters XBridge Shield RP18 column (3.0 × 100 mm, 2.5 *μ*m), XSelecT HSS T3 column (3.0 × 100 mm, 2.5 *μ*m), and Agilent Zorbax SB-C18 RRHT Threaded Column (3.0 × 150 mm, 5 *μ*m) were tested (separate chromatogram is shown in Figure 5), and eventually XSelecT HSS T3 column (3.0 × 100 mm, 2.5 *μ*m) was chosen due to the good separation and peak shapes for all the four analytes. Compared with the existing LC-MS/MS methods, the established method in our study achieved proper retention times, reduced peak width, and symmetric peak shape.

### 4.2. Sample Pretreatment

Samples were pretreated to remove proteins and other potential interferents and to extract the analytes from serum. Several methods are usually used for sample pretreatment, including solid-phase extraction, liquid-liquid extraction, and protein precipitation. Solid-phase extraction and liquid-liquid extraction were excluded due to lower recoveries, complexity, and being time-consuming and of high cost. As for the choice of protein precipitant, methanol, acetonitrile, and methanol containing 1% formic acid were all tested. Finally, we found that pure acetonitrile precipitation achieved the highest purification as well as the most satisfying extraction recovery, which did not result in any ion suppression or enrichment.

### 4.3. Choice of Internal Standards

Isotopic internal standards (RIF-D8, PZA-D3, INH-D4, and EMB-D4) were used in this study. Most published studies chose analogues or homologues, like RFX and PFM, as internal standards, considering their structural similarities to the parent compounds. However, potential shortcomings exist. For example, concomitant use of these analogous or homologous agents for patients might interfere with the detection when these analogues or homologues are used as internal standards. The ability of analogues to compensate for degradation and loss of the analytes during sample preparation is not as good as that of the isotopic internal standards. Though a few studies selected isotopic compounds as internal standards, RFP-D3 was used for RFP [16–19]. However, in our study, RFP-D3 showed a high level in standards of RFP (about 10%) and in serum samples of patients treated with RFP by chromatography, suggesting that RFP-D3 would interfere with the quantification of RFP. In our study, RFP-D3 was originally replaced by RFP-D8 to serve as an internal standard.

### 4.4. Stability of Isoniazid

Three analytes (PZA, RFP, and EMB) were stable when stored in different conditions, including room temperature for 4 h, 10°C for four days, −70°C for 30 days, and three cycles of freezing-thawing. These results indicated that these three analytes were generally stable during storage, handling, and analysis of the samples. However, analyte degradation was found when INH was placed at room temperature for 4 h or even 2 h. According to our study, once the isoniazid sample was collected, it should be placed in an ice bath and analyzed or stored in −70°C within 2 h.

### 4.5. Application to Clinical Samples

We applied the established LC-MS/MS method to monitor serum concentrations of the four antituberculosis drugs at 0 h, 2 h, and 6 h for eight tuberculosis patients. According to our results, all the patients reached the highest concentration at 6 h, which deviated from previous reporting that *C*_max_ of the four first-line antituberculosis reached between 2 h and 4 h [9]. Thus, better designed studies with larger population and more data points were urgent to confirm the peak time in Chinese patients and explore the possible explanations. Serum drug concentrations of the eight patients were all below the recommended targeting concentrations for the four antituberculosis drugs (*C*_max_ normal range of RFP: 8–24 *μ*g/mL; INH: 3-6 *μ*g/mL; PZA: 20–60 *μ*g/mL; EMB: 2-6 *μ*g/mL) [20], suggesting further studies were warranted to explore whether the current targeting concentrations were proper for tuberculosis patients or whether drug dosages should be adjusted to the targeting levels to improve the treatment response.

### 4.6. Novelty of the Developed Method

Compared with previous studies [10–19], HPLC column and mobile phase, together with gradient elution applied in this study, significantly improved the peak shapes. The total volume for sample analyzing was only 50 *μ*L, which was much lower than the reported method. In addition, the one-step protein precipitation method for sample treatment was also simpler than the reported two-step liquid-liquid extraction and solid-phase extraction methods. Besides, in this study, we employed isotopic-labeled internal standards to adjust for the loss and degradation of the analytes during pretreatment, especially that RFP-D8, instead of RFP-D3, was used for the first time. This method has been successfully applied to clinical practice for TDM of the first-line anti-TB drugs, which is also attractive for the pharmacokinetic analysis of these drugs, because of its simple sample processing steps, high analytical sensitivity, and accuracy.

## 5. Conclusion

In this study, a simple, rapid, and robust LC-MS/MS method has been developed and validated for simultaneous quantification of four first-line anti-TB drugs in human serum. This method has been successfully applied to clinical practice for TDM of the first-line anti-TB drugs.

## Figures and Tables

**Table 1:** Optimized compound-dependent MS parameters for detection of the analytes and ISs.

Compound	Precursor ion (*m*/*z*)	Product ion (*m*/*z*)	DP/*V*	CE/*V*	CXP/*V*
INH	138.0	121.0	50.0	18.0	10.0
RFP	823.1	791.4	100.0	13.0	14.0
EMB	205.5	116.1	50.0	40.0	10.0
PZA	124.1	81.0	50.0	25.0	6.0
INH-D4	142.0	125.1	50.0	21.0	10.0
RFP-D8	831.5	799.6	92.0	15.0	15.0
EMB-D4	209.3	120.1	50.0	21.0	10.0
PZA-D3	127.2	84.1	60.0	24.0	6.0

PZA: pyrazinamide, INH: isoniazid, RFP: rifampicin, EMB: ethambutol, DP: declustering potential, CE: collision voltage, CXP: collision cell exit potential, IS: internal standard, and MS: mass spectrum.

**Table 2:** Regression equation, correlation coefficient, linearity range, and LLOQ of each analyte.

Analytes	Regression equation	Correlation coefficient (*r*)	Linearity range (*μ*g/mL)	LLOQ (*μ*g/mL)
PZA	*Y*_PZA_ = (0.2423 ± 0.0006) *x* + (0.0230 ± 0.0046)	0.9992 ± 0.0004	1.02∼60.0	1.02
INH	*Y*_INH_ = (0.6607 ± 0.0323) *x* + (0.0090 ± 0.0071)	0.9991 ± 0.0004	0.152∼10.0	0.152
EMB	*Y*_EMB_ = (0.3503 ± 0.0012) *x* + (0.0027 ± 0.0015)	0.9995 ± 0.0003	0.0998∼5.99	0.0998
RFP	*Y*_RFP_ = (0.1839 ± 0.1354) *x* + (0.0185 ± 0.0046)	0.9982 ± 0.0020	0.500∼30.0	0.500

PZA: pyrazinamide, INH: isoniazid, RFP: rifampicin, EMB: ethambutol, and LLOQ: lower limit of qualification.

**Table 3:** Precision and accuracy of the method at four concentration levels.

Analytes	QC levels	Referred value (*μ*g/mL)	RSD (%)		Accuracy (%)	
			Intrarun (*n* = 6)	Interrun (*n* = 6×3)	Intrarun (*n* = 6)	Interrun (*n* = 6×3)
PZA	LLOQ	1.02	8.06	8.33	98.43	100.00
	Low	1.60	4.21	3.71	94.92	95.32
	Medium	16.0	4.59	3.46	98.38	98.46
	High	48.0	2.68	3.64	99.63	100.66

INH	LLOQ	0.152	4.69	6.70	95.02	98.68
	Low	0.268	2.98	4.62	91.13	94.67
	Medium	2.68	4.20	4.05	97.63	99.58
	High	8.04	2.53	3.92	99.63	100.81

EMB	LLOQ	0.0998	5.13	5.39	97.80	100.20
	Low	0.161	2.74	3.93	91.67	94.00
	Medium	1.61	12.46	8.49	103.82	99.58
	High	4.60	3.79	3.17	104.62	104.02

RFP	LLOQ	0.500	5.00	7.27	90.15	98.00
	Low	0.810	4.26	5.87	94.03	99.26
	Medium	8.100	8.73	6.43	95.70	97.77
	High	24.300	3.42	3.39	96.23	95.13

PZA: pyrazinamide, INH: isoniazid, RFP: rifampicin, EMB: ethambutol, QC: quality control, RSD: relative standard derivation, and LLOQ: lower limit of qualification.

**Table 4:** Recovery and matrix effect of four analytes in QC sample at three levels (*n* = 6).

Analytes	QC levels	Recovery (%)	Matrix effect
PZA	Low	87.04	0.99 ± 0.08
	Medium	91.25	0.99 ± 0.04
	High	90.80	1.01 ± 0.05

INH	Low	79.24	0.87 ± 0.04
	Medium	89.56	0.92 ± 0.05
	High	81.68	0.95 ± 0.03

EMB	Low	83.35	0.96 ± 0.04
	Medium	87.46	1.00 ± 0.03
	High	86.58	1.01 ± 0.04

RFP	Low	95.01	0.97 ± 0.12
	Medium	89.70	1.05 ± 0.06
	High	94.16	0.99 ± 0.05

PZA: pyrazinamide, INH: isoniazid, RFP: rifampicin, EMB: ethambutol, and QC: quality control.

## Data Availability

The data used to support the findings of this study are included within the article.
